# Gustave Roussy Immune Score as a Novel Prognostic Scoring System for Colorectal Cancer Patients: A Propensity Score Matching Analysis

**DOI:** 10.3389/fonc.2021.737283

**Published:** 2021-11-30

**Authors:** Shan Tian, Yinghao Cao, Yanran Duan, Qi Liu, Pailan Peng

**Affiliations:** ^1^ Department of Infectious Diseases, Union Hospital, Tongji Medical College, Huazhong University of Science and Technology, Wuhan, China; ^2^ Department of Colorectal Surgery and Gastroenterology, Wuhan Union Hospital, Tongji Medical College of Huazhong University of Science and Technology, Wuhan, China; ^3^ Department of Epidemiology and Biostatistics, School of Public Health, Tongji Medical College, Huazhong University of Science and Technology, Wuhan, China; ^4^ Department of Gastroenterology, The Affiliated Hospital of Guizhou Medical University, Guiyang, China

**Keywords:** colorectal cancer, Gustave Roussy Immune Score, survival analysis, predictive value, propensity score matching

## Abstract

**Aim:**

The Gustave Roussy Immune Score (GRIm-Score) was originally designed to select cancer patients for immunotherapy, and later was reported to be a novel prognostic scoring system in lung cancer and esophageal cancer. This study was aimed to determine the prognostic role and predictive performance of GRIm-Score in colorectal cancer (CRC) CRC patients.

**Methods:**

We conducted a single-institution study of 1,579 adult CRC patients receiving surgical removal, and those patients were divided into low GRIm-Score group (scores 0, 1) and high GRIm-Score group (scores 2, 3). Propensity score matching (PSM) was executed to balance the potential confounding factors between the two groups. Survival and time-dependent receiver operating characteristic (Td-ROC) analyses were applied to depict the prognostic role and predictive significance of GRIm-Score in CRC patients.

**Results:**

There were 200 cases CRC patients in high GRIm-Score group and 1,379 cases in low GRIm-Score group. CRC patients with high GRIm-Score correspond with higher level of CEA, CA125, and inflammatory indexes, such as NLR, PLR, SII, PNI, and ALRI. Correlation analysis exhibited that GRIm-Score correlated well with the established inflammatory indexes. Survival analysis revealed that CRC patients in high GRIm-Score group showed worse overall survival (OS, P <0.0001) and disease-free survival (DFS, P <0.0001) compared with those in low GRIm-Score group. Results from multivariate Cox regression implicated that high GRIm-Score was not only a potent prognostic index for unfavorable OS (HR = 1.622, 95%CI: 1.118–2.355, P = 0.0109), but also a potent risk factor for worse DFS (HR = 1.743, 95%CI: 1.188–2.558, P = 0.0045). Td-ROC analysis demonstrated that GRIm-Score exhibited the superior discriminatory power in the prediction of OS and DFS when compared to SII, PNI, and ALRI. Such strong associations between high levels of preoperative GRIm-Score and unfavorable survival outcomes remained robust after PSM analysis.

**Conclusion:**

GRIm-Score, a novel inflammatory and nutritional risk scoring system, is a potent prognostic index in CRC patients receiving surgical removal. GRIm-Score can be used as an effective and simplified risk stratification tool for postoperative survival prediction of CRC patients.

## Introduction

Colorectal cancer (CRC) is still one of the most common malignant neoplasms of the digestive tract (1). CRC is second in terms of mortality (9.2%), and it is estimated that the total number of deaths from colon cancer and rectal cancer will increase by 71.5 and 60%, by the year 2035, respectively (2). Surgical resection is still viewed as the most preferred option for the treatment of CRC. In spite of great advances in surgical techniques and medical care strategies, the long-term prognosis of CRC individuals still remains an area of improvement (3). Gaining deep insights into the prognostic biomarkers will be very useful for oncologists and surgeons to precisely identify the potential patients with higher probability of unfavorable outcomes, and thus settle a personalized treatment plan.

CRC is a kind of heterogeneous disease occurred in the intestinal epithelium, and is characterized by the dysregulated immune response (4). Emerging evidences demonstrate that cancer-associated inflammation is a main mechanism to promote the progression and deterioration of CRC. Inflammatory response in tumor microenvironments also could exert great impacts on tumor metastasis and immunity, representing a key direction with regard to anti-tumor treatment (5). Systemic inflammatory indexes based on leukocytes, such as lymphocyte to monocyte ratio (LMR), platelet to lymphocyte ratio (PLR) (6), and neutrophil to lymphocyte ratio (NLR) have been reported in several malignant cancers as prognostic markers. Moreover, high systemic inflammatory response assessed by other scoring systems, such as systemic immune-inflammation index (SII) (7), prognostic nutritional index (PNI) (7), and aspartate aminotransferase–lymphocyte ratio index (ALRI) (8) have also been proven to be associated with unfavorable survival in patients with CRC. These findings attract attention to create a novel risk scoring system that provides clinicians with objective information for the prognostic prediction and risk stratification.

The Gustave Roussy Immune Score (GRIm-Score) was originally proposed by Bigot and corworkers (9) in 2017 as an objective risk score to optimize the selection of eligible participants testing new immune-checkpoint therapies (ICTs) in phase I clinical trials. GRIm-Score is based on serum lactate dehydrogenase, NLR and serum albumin, and proven to be a potent prognostic index associated with the overall survival (OS) of cancer patients. The prognostic significance of preoperative GRIm-Score also has been validated in early-stage non-small-cell lung cancer (10–12), small cell lung cancer (13), and esophageal squamous cell carcinoma (14). However, little is known whether the high GRIm-Score is remarkably associated with less favorable outcomes in patients with CRC. Hence, this retrospective study was designed to depict the clinical significance and prognostic value of GRIm-Score in individuals with CRC, and to determine the predictive performance of GRIm-Score for survival rate. Moreover, we also exploited the propensity score matching (PSM) to reduce potential confounding factors in our analysis.

## Methods

### Study Population

This retrospective clinical study was conducted on the single medical center from the Wuhan Union hospital. All relevant procedures were prospectively reviewed and approved by the Clinical Research Ethics Committee of Wuhan Union hospital (No. 2018-S377), and this investigation was conducted in line with the Helsinki Declaration. The inclusion criteria were listed as follows: (1) CRC patients experiencing surgical removal in our institution; (2) The diagnosis of CRC was confirmed by histopathology; and (3) CRC individuals were with complete follow-up data. Whereas, the exclusion criteria were also listed as follows: (1) CRC individuals with unknown clinical group stage; (2) Those patients who received intestinal resection outside of our institution; (3) Those patients who received systemic chemoradiotherapy prior to surgical resection; and (4) CRC patients were concomitant with acute infectious diseases during this hospitalization. A total of 3,500 CRC patients from our medical center were initially screened, and 1,579 cases of CRC patients underwent surgical removal were finally included in this clinical analysis based on the above criteria. Among them, 1,399 patients received radical excision and 180 cases received palliative surgery.

### Data Collection

**Figure 1:**
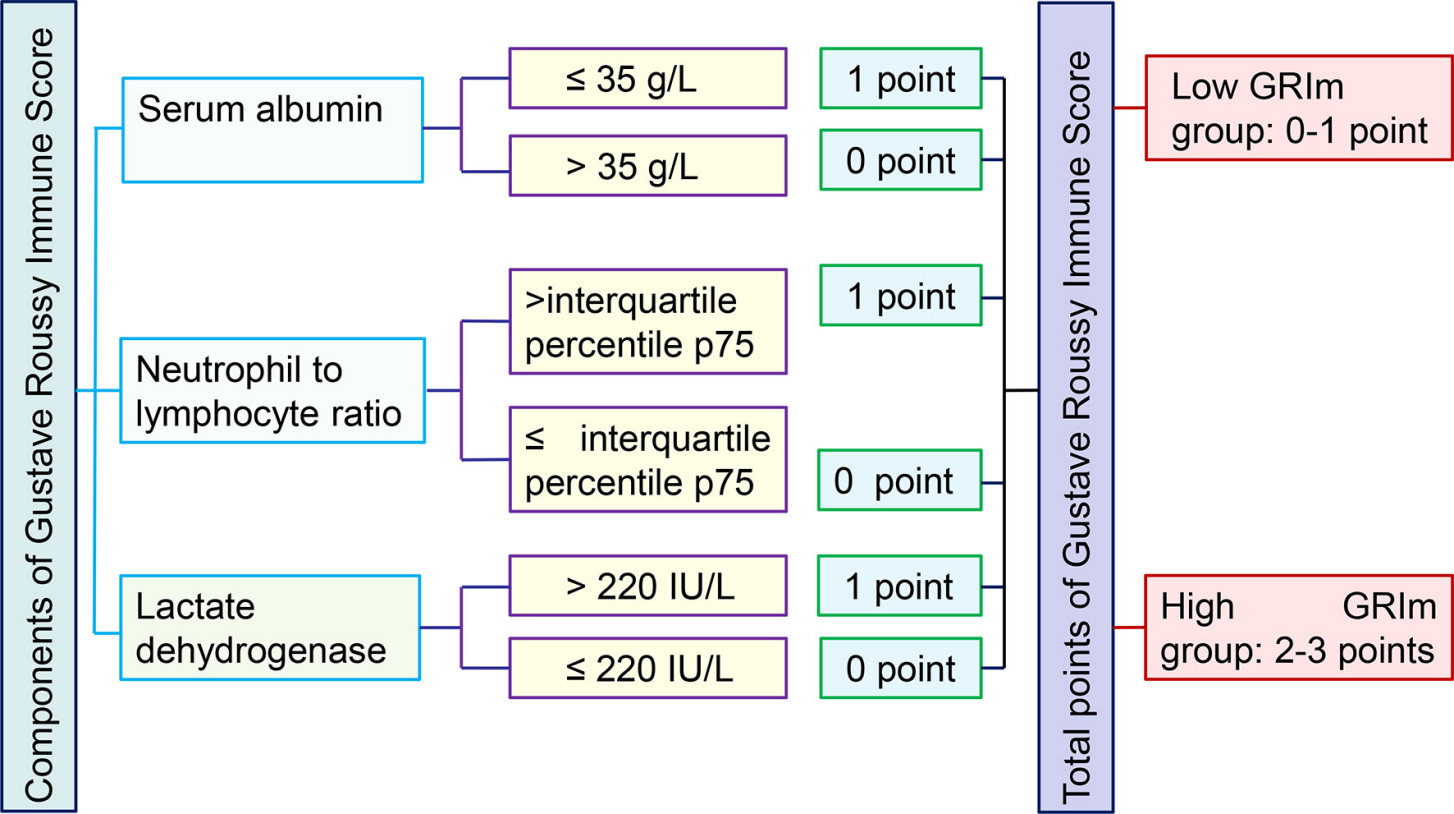
The detailed definition and grouping items of GRIm-Score.

The demographical indexes (age, sex, family history of cancer, body mass index, and smoke), tumor features (tumor site, tumor size, tumor differentiation, T stage, N stage, TNM stage, chemotherapy, and radiotherapy), and laboratory markers were retrospectively collected from our medical records. Blood sampling was collected within three days before surgical removal. The following serum indexes were collected for this analysis: carcinoembryonic antigen (CEA), CA724, CA199, CA125, AST, ALT, albumin (ALB), prealbumin (PAB), total protein (TP), direct bilirubin (DBIL), total bilirubin (TBIL), GGT, alkaline phosphatase (ALP), serum lactate dehydrogenase (LDH), total bile acid (TBA), uric acid (UA), serum creatinine, and blood urea nitrogen (BUN). Serum ALB, TP, and PAB were converted into dichotomous based on the lower limit of normal, while other serums markers were changed into binary variables based on the upper of normal. Moreover, we also assessed several frequently-used inflammatory variables, such as neutrophil to lymphocyte ratio (NLR), platelet to lymphocyte ratio (PLR), systemic immune-inflammation index (SII), aspartate aminotransferase to lymphocyte ratio (ALRI), and prognostic nutritional index (PNI). These combined indexes were also converted into dichotomous variables based on the median value. The most important index (GRIm-Score) was formulated by serum LDH, NLR, and serum albumin. We divided the 1,579 cases of CRC patients into high GRIm-Score group (scores 2, 3) and low GRIm-Score group (scores 0, 1) (10). The detailed definition and grouping criteria of GRIm-Score was vividly shown in **Figure 1**. We obtained the survival information through telephone or outpatient follow-up. The last follow-up time was Dec. 31, 2019. We defined the period from surgery to death or last follow-up time as OS, and the interval from surgery to recurrence as disease-free survival (DFS).

### PSM Analysis

Given that the classification of two groups (high GRIm-scroe group vs. low GRIm-Score group) was really not randomized, unbalanced variables might lead to selection bias. Therefore, PSM analysis was applied to reduce the potential selection bias. We initiated a 1:3 (high GRIm-Score group vs. low GRIm-Score group) matched analysis by PSM with a nearest-neighbor matching algorithm to adjust the baseline characteristic differences between the two groups. The PSM analysis lead to a balanced cohort including high GRIm-Score group (N = 198) and low GRIm-Score group (N = 557). Significantly, all potential confounders must not be affected by any component of GRIm-Score in the PSM model (10), indicating that any peripheral indexes obtained from biochemistry tests or blood routine would not be suitable for PSM balance. After PSM, baseline clinic-pathologic features were well-balanced between high and low GRIm-Score groups.

### Statistical Analysis

As we converted most continuous variables into categorical variables, so these data were presented with number and percentage, and analyzed with Chi-square or Fisher exact test. Spearman correlation analysis was conducted to rate the potential relationship between GRIm-Score and several combined inflammatory indexes (NLR, PLR, SII, PNI, and ALRI) as well as tumor markers (CEA, CA-125). The areas under the curve (AUC) for GRIm-Score, SII, PNI, and ALRI were measured and compared with the time dependent receiver operating characteristic (Td-ROC) curves. The median survival time was assessed by the Kaplan–Meier method, and survival distributions between two groups were compared with the log-rank test. Univariate and multivariate Cox regression models were exploited to depict the association between high levels of GRIm-Score and risk of death or recurrence among CRC patients. Statistical analyses were completed with MedCal (version 19.1.3) and STATA (version 16.0); the significance level of statistical analysis was set at 0.05.

## Results

### Basic Clinical Information

Based on related inclusion/exclusion criteria, 1,579 cases of CRC patients who received surgical removal were finally included in this analysis. There were 941 (59.6%) male patients and 638 (40.4%) female. The mean age of included CRC individuals was 58.11 years old with the range of 21 to 85. After operation, 842 individuals were treated with chemotherapy, and only 86 cases received radiotherapy. According to GRIm-Score system, there were 961 patients (60.86%) reached a score of 0, 418 patients (26.47%) reached a score of 1,176 patients (11.15%) reached a score of 2, and 24 patients (1.52%) reached a score of 3, respectively. We initially divided GRIm score into four groups (scores 0, 1, 2, and 3), and we analyzed the correlation of GRIm score with clinical features (**Table S1**). We also performed the Cox regression (**Tables S2, S3**) to explore the prognostic role of four-category GRIm-Score (**Figure S1**). However, we found that the survival outcomes of CRC patients with score 1 were not significantly different from CRC patients with score 2 (**Figure S2**), indicating that worse prognosis of CRC patients did not increase gradually with GRIm score, especially in scores 1 and 2. So, we still divided patients into a high GRIm score group (scores 2 and 3) and low GRIm score (scores 0 and 1), which was commonly used in another two clinical research studies (9, 14). In total, 200 individuals (12.67%) with CRC were divided into the high GRIm-Score group, 1,379 individuals (87.33%) were divided into the low GRIm-Score group. As listed in **Table S4**, CRC patients with high GRIm-Score corresponds with higher level of CEA, CA125, and inflammatory indexes, such as NLR, PLR, SII, PNI, and ALRI.

### Correlation Analysis

**Figure 2:**
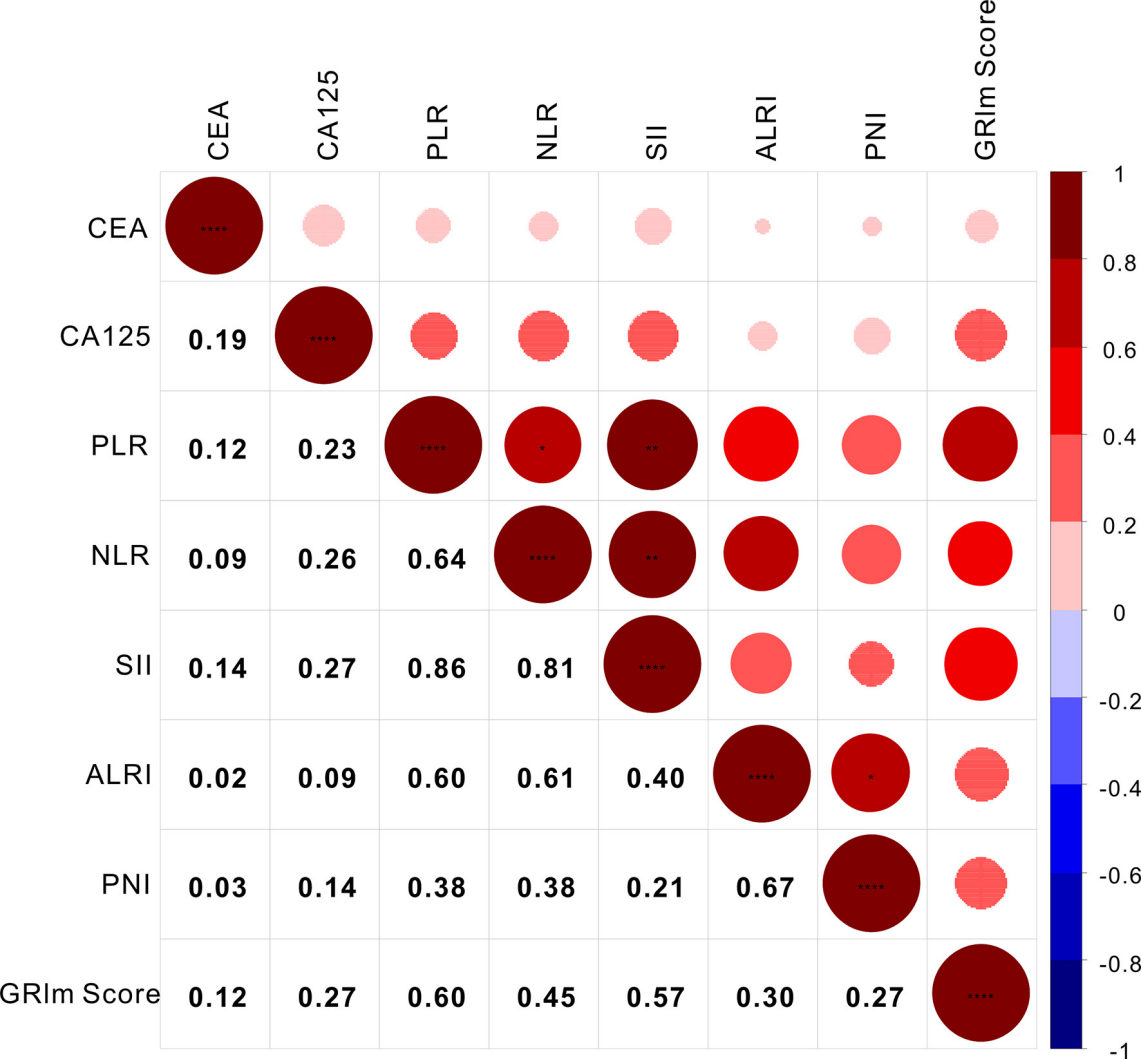
Correlation analysis of GRIm-Score with inflammatory scores and tumor markers. * stands for P<0.05, ** stands for P<0.01, ** *stands for P<0.001.

As a novel inflammatory score, it is necessary to assess the correlation of GRIm-Score with other well-established inflammatory indexes, such as SII, PNI, ALRI, NLR, and PLR. Therefore, Spearman correlation analysis was executed to rate the relationship GRIm-Score showed strong association with the well-established inflammatory indexes. As shown in **Figure 2**, GRIm-Score correlated well with PLR (r = 0.60, P <0.0001), NLR (r = 0.45, P <0.0001), SII (r = 0.57, P <0.0001), ALRI (r = 0.30, P <0.0001) and PNI (r = 0.27, P <0.0001). Moreover, as the chi-square analysis revealed that CRC patients with high GRIm-Score correspond with higher level of CEA, CA125, so we also measured the association between GRIm-Score and tumor markers. We also noticed the positive correlation of GRIm-Score with CEA (r = 0.12, P <0.0001) and CA125 (r = 0.27, P <0.0001).

### Survival Outcomes

**Figure 3:**
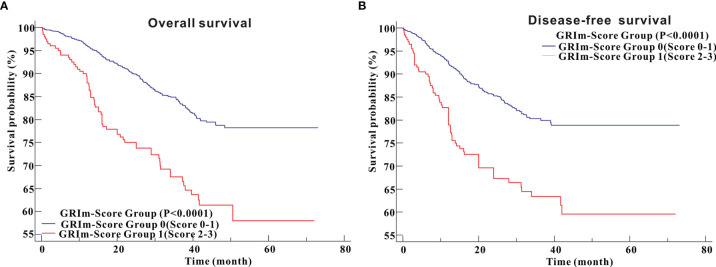
Survival analysis of CRC patients stratified based on GRIm-Score before propensity score matching. **(A)** Overall survival; **(B)** Disease-free survival.

Kaplan–Meier survival analysis revealed that CRC patients in high GRIm-Score group showed worse OS (P <0.0001) compared with those in low GRIm-Score group (**Figure 3A**). Similarly, survival analysis also indicated that CRC patients in high GRIm-Score group experienced shorter DFS time (P <0.0001) compared with those in low GRIm-Score group (**Figure 3B**). In addition, the proportion of chemotherapy was significantly different between low GRIm-Score group and high GRIm-Score group (P = 0.0047), and we conducted the subgroup analysis based on the presence of chemotherapy. Subgroup analysis exhibited that GRIm-Score was a potent prognostic index, not only in CRC patients receiving postoperative chemotherapy, but also in patients without chemotherapy (**Figure S3**). We further employed univariate Cox regression along with multivariate regression to determine the independent risk variables of CRC patients. As displayed in **Table 1**, high GRIm-Score was not only a potent prognostic index for unfavorable OS (HR = 1.622, 95%CI: 1.118–2.355, P = 0.0109), but also a potent risk factor for worse DFS (HR = 1.743, 95%CI: 1.188–2.558, P = 0.0045).

**Table 1:** Multivariate cox analysis of CRC patients in the whole cohort.

Clinical feature		OS HR&95%CI	P value	DFS HR&95%CI	P value
Smoke	No	**Reference**		**Reference**	
	Yes	0.563 (0.358–0.885)	0.0127	0.613 (0.381–0.984)	0.0428
Chemotherapy	No	**Reference**			
	Yes	0.575 (0.416–0.795)	0.0008		
CEA	Normal	**Reference**			
	High	1.652 (1.180–2.314)	0.0034		
AST	Normal			**Reference**	
	High			2.154 (1.205–3.850)	0.0096
PAB	Normal	**Reference**		**Reference**	
	High	1.651 (1.170–2.330)	0.0043	1.528 (1.094–2.135)	0.013
TNM stage	I			**Reference**	
	II			0.784 (0.257–2.397)	0.67
	III			1.534 (0.504–4.672)	0.4514
	IV			5.722 (1.908–17.162)	0.0019
T stage	I	**Reference**		**Reference**	
	II	0.730 (0.243–2.195)	0.575	1.021 (0.303–3.438)	0.9731
	III	2.478 (0.917–6.697)	0.0737	3.176 (1.112–9.068)	0.0309
	IV	3.930 (1.427–10.822)	0.0081	3.478 (1.191–10.152)	0.0226
N stage	0	**Reference**		**Reference**	
	1	1.871 (1.060–3.304)	0.0307	1.246 (0.749–2.071)	0.397
	2	2.834 (1.626–4.941)	0.0002	1.866 (1.159–3.004)	0.0102
GRIm-Score	Score 0–1	**Reference**		**Reference**	
	Score 2–3	1.622 (1.118–2.355)	0.0109	1.743 (1.188–2.558)	0.0045

CEA, carcinoembryonic antigen; AST, aspartate aminotransferase; PAB, prealbumin; GRIm-Score, Gustave Roussy Immune Score; OS, overall survival; DFS, disease-free survival.

### Predictive Performance of GRIm-Score

**Figure 4:**
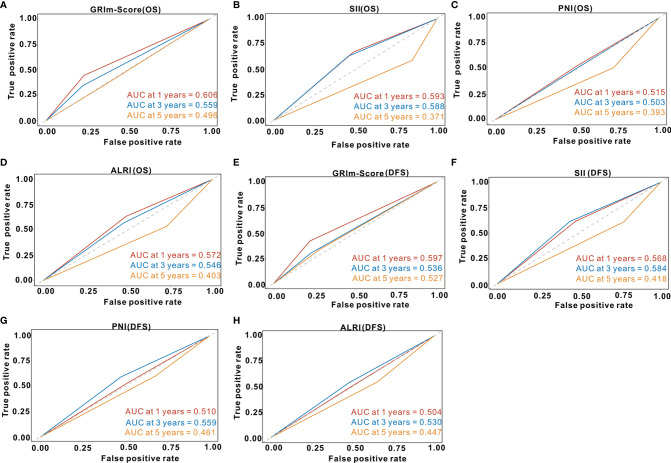
Time-dependent receiver operating curves of GRIm-Score, SII, PNI and ALRI for the prediction of postoperative survival rate in the whole cohort. **(A)** GRIm-Score (Overall survival); **(B)** SII (Overall survival); **(C)** PNI (Overall survival); **(D)** ALRI (Overall survival); **(E)** GRIm-Score (Disease-free survival); **(F)** SII (Disease-free survival); **(G)** PNI (Disease-free survival); **(H)** ALRI (Disease-free survival). SII, systemic immune-inflammation index; PNI, prognostic nutritional index; ALRI, aspartate aminotransferase to lymphocyte ratio.

We utilized td-ROC analysis to assess the predictive capability of GRIm-Score for survival rate of CRC patients. As shown in **Figure 4A**, the overall performance of GRIm-Score for the prediction of 1-year, 3-year, and 5-year OS rate was 0.606, 0.559, and 0.496, respectively. Then, we also compared the predictive performance of GRIm-Score with other combined indexes, such as SII, PNI, and ALRI. Encouragingly, GRIm-Score held superior predictive accuracy for OS rate in CRC patients to the above combined inflammatory indexes (**Figures 4B–D**). Additionally, the overall performance of GRIm-Score, as measured by AUC, for the prediction of 1-year, 3-year, and 5-year DFS rate was 0.597, 0.536, and 0.527, respectively (**Figure 4E**). GRIm-Score also held higher AUC for the prediction of OS rate in CRC patients than any single combined inflammatory indexes (**Figures 4F–H**).

### Survival Outcomes After PSM

We noticed that there existed significant difference of family history of cancer, BMI, tumor site, T stage, TNM stage, and tumor site between high and low GRIm-Score groups. Accordingly, we exploited 1:3 PSM analysis to balance those confounding bias between high GRIm-Score group (N = 198) and low GRIm-Score group (N = 557). Approximately 369 patients (48.87%) scored 0, 188 patients (24.90%) score 1 point, 174 patients (23.05%) scored 2 points, and 24 patients (3.18%) score 3 points, respectively. As exhibited in **Table S5**, all of the estimated indexes were adequately balanced between high and low GRIm-Score groups after PSM.

**Figure 5:**
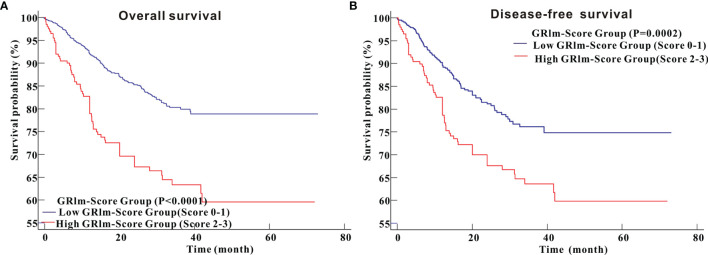
Kaplan–Meier curves for overall survival and disease-free survival between high GRIm-Score group and low GRIm-Score group in propensity-matched cohorts. **(A)** Overall survival; **(B)** Disease-free survival.

In the PSM cohort, Kaplan–Meier survival analysis indicated that CRC patients in high GRIm-Score group possessed more unfavorable OS (P <0.0001) than CRC patients in low GRIm-Score group (**Figure 5A**). Similarly, survival analysis in the PSM cohort also demonstrated that CRC patients in high GRIm-Score group experienced shorter DFS time (P = 0.0002) compared with those in low GRIm-Score group (**Figure 5B**). Univariate Cox regression along with multivariate regression was further applied to identify the independent risk variables of CRC patients. As displayed in **Table 2**, high GRIm-Score was not only a strong prognostic index for unfavorable OS (HR = 1.811, 95%CI: 1.218–2.691, P = 0.0033), but also a strong risk factor for worse DFS (HR = 2.121, 95%CI: 1.417–3.174, P = 0.0003) after adjustment for several potential covariates.

**Table 2:** Multivariate cox analysis of CRC patients in propensity-matched cohort.

Clinical feature		OS HR&95%CI	P value	DFS HR&95%CI	P value
Smoke	No	Reference		–	–
	Yes	0.502 (0.277–0.907)	0.0224	–	–
T stage	T1	Reference		–	–
	T2	0.274 (0.078–0.961)	0.0432	–	–
	T3	0.802 (0.286–2.254)	0.6762	–	–
	T4	1.519 (0.537–4.294)	0.4308	–	–
TNM stage	I	–	–	Reference	
	II	–	–	6.480 (0.871–48.200)	0.068
	III	–	–	12.387 (1.699–90.29)	0.013
	IV	–	–	50.10 (6.817–368.206	0.0001
Chemotherapy	No	Reference		–	–
	Yes	0.625 (0.418–0.935)	0.0222	–	–
CEA (ug/L)	Normal	–	–	Reference	
	High	–	–	1.600 (1.063–2.407)	0.0243
DBIL(umol/L)	Normal	–	–	Reference	
	High	–	–	1.894 (1.045–3.436)	0.0354
GGT(U/L)	Normal	Reference		–	–
	High	2.084 (1.306–3.325)	0.0021	–	–
GRIm-Score	Low (score 0,1)	Reference		Reference	
	High (score 2,3)	1.811 (1.218–2.691)	0.0033	2.121(1.417~3.174)	0.0003

CEA, carcinoembryonic antigen; DBIL, direct bilirubin; GGT, glutamyl transpeptidase; GRIm-Score, Gustave Roussy Immune Score; OS, overall survival; DFS, disease-free survival.

### Predictive Performance of GRIm-Score After PSM

**Figure 6:**
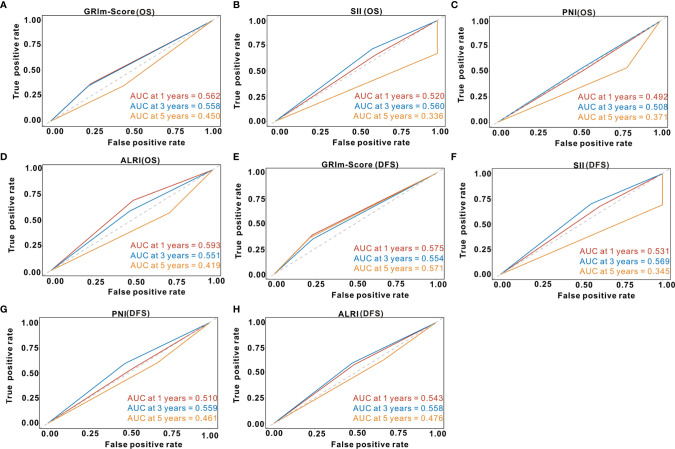
Time-dependent receiver operating curves of GRIm-Score, SII, PNI, and ALRI for the prediction of postoperative survival rate in propensity-matched cohort. **(A)** GRIm-Score (Overall survival); **(B)** SII (Overall survival); **(C)** PNI (Overall survival); **(D)** ALRI (Overall survival); **(E)** GRIm-Score (Disease-free survival); **(F)** SII (Disease-free survival); **(G)** PNI (Disease-free survival); **(H)** ALRI (Disease-free survival). SII, systemic immune-inflammation index; PNI, prognostic nutritional index; ALRI, aspartate aminotransferase to lymphocyte ratio.

Td-ROC analysis was also performed to evaluate the predictive ability of GRIm-Score for OS rate among CRC patients from the PSM cohort. As shown in **Figure 6A**, the overall predictive performance of GRIm-Score for predicting 1-year, 3-year, and 5-year OS rate was 0.562, 0.558, and 0.450, respectively. Then, we also compared the predictive performance of GRIm-Score with other commonly-used combined indexes, such as SII, PNI, and ALRI, in the PSM cohort. Encouragingly, GRIm-Score held superior predictive accuracy for DFS rate in CRC patients to the above combined inflammatory indexes (**Figures 6B–D**). Moreover, the overall performance of GRIm-Score, as assessed by AUC, for predicting 1-year, 3-year, and 5-year DFS rate was 0.575, 0.554, and 0.571, respectively (**Figure 6E**). GRIm-Score also held better performance for the prediction of DFS rate in CRC patients than any single combined inflammatory indexes (**Figures 6F–H**).

## Discussion

Inflammation is an acquainted hallmark of malignancies that greatly contributes to the occurrence and progression of most cancers. In CRC, there is an increasing evidence for the role that systemic inflammation plays in deterioration and progression of cancer, and undesirable survival (5, 8). Recently, with rapid technological advances in molecular biology and biochemistry, several risk scoring systems based on inflammatory and nutritional indexes have been developed for the prediction of prognosis in CRC patients (7, 15). Among these risk scoring systems, GRIm-Score is a novel predictive model which has never been studied in CRC individuals. This present study was the first literature to systematically report the prognostic significance of the GRIm-Score, which covered combined effects of LDH, ALB, and NLR, for precisely predicting both OS and DFS of postoperative CRC patients. Based on 1,579 CRC individuals, we could conclude that CRC patients in high GRIm-Score group exhibited shorter OS and DFS time compared with those in low GRIm-Score group. Such strong associations between preoperative GRIm-Score and survival outcomes were still remained when validated by PSM analysis.

GRIm-Score is composed of both inflammatory and nutritional conditions by effectively incorporating LDH, ALB, and NLR, all of which have been widely explored to reflect systemic inflammation and malnutrition of CRC patients (16–22). Feng et al. (18) conducted a meta-analysis included 1,219 CRC patients and found that high levels of serum LDH were significantly correlated with undesirable OS and PFS. A recent meta-analysis performed by Li et al. (23) revealed that preoperative NLR was a very effective biomarker for the prediction of outcomes (OS and recurrent-free survival and DFS) in patients with CRC. A recent clinical trial demonstrated that reduced serum ALB was an independent risk index for unfavorable OS in patients with metastatic CRC (24). Due to the strong correlation with clinical outcomes, the combined index (GRIm-Score) was surely more powerful than any single index (LDH, ALB or NLR). Moreover, as the three indexes are easily accessible with low cost in the clinical practice, we proposed that GRIm-Score should be utilized for survival prediction.

GRIm-Score is a relatively new scoring system put forward by Bigot et al. (9), and validated only in the following three clinical trials. Feng et al. (14) conducted a retrospective study including 372 ESCC patients receiving surgical resection. They found that ESCC patients in high GRIm-Score group experienced more inferior cancer-specific survival than those in low GRIm-Score group. The GRIm-Score, instead of single NLR, LDH or ALB, was proven to be an independent risk factor for CSS *via* multivariate Cox regression. In addition, a recent study also investigated the prognostic role of GRIm-Score in patients with small cell lung cancer. The results indicated that OS time was significantly shorter in high GRIm-Score group than in low GRIm-Score group, but no significant difference was noticed for PFS. Moreover, Li et al. (10) assessed the potential prognostic role of GRIm-Score in 405 NSCLC patients with stages I–II receiving video-assisted thoracoscopic surgery lobectomy by PSM analysis. They reported that strong correlations between high levels of GRIm-Score assessed by 3-category risk scale and unfavorable survival outcomes still existed after PSM analysis. They also discovered that GRIm-Score exhibited the superior discriminatory power to other peripheral blood biomarkers for predicting survival rates of NSCLC patients with stages I–II. Similar to their analysis, the potent prognostic significance of GRIm-Score for survival existed not only in the entire cohort but also in the PSM cohort.

Inflammation-based indexes are easily obtained from the routinely detected laboratory results and could be conveniently used to reflect the systemic inflammatory status. PNI, determined by the albumin level and lymphocyte count, is regarded as the reflection of the immuno-nutritious status. PNI was reported to be equally as important as rating TNM stage in the management of CRC patients (25). SII was investigated as a potent prognostic index in several types of malignant tumors, including CRC (26, 27). Increased levels of SII indicates high platelets, neutrophils but low lymphocytes (28). A clinical study with 355 CRC patients evaluated the correlation between PNI and SII (29), and levels of PNI were positively associated with SII. Consistent with their findings, we analysis also confirmed the strong relationship between PNI and SII (r = 0.21, P <0.001). Compared with these well-established inflammatory scores, GRIm-Score is an emerging grouping score, and also exhibited well predictive performance of survival outcomes among CRC patients. Encouragingly, td-ROC analyses also implicated that GRIm-Score exhibited the superior discriminatory power to other peripheral blood biomarkers, such as SII, PNI, and ALRI, for predicting survival rates of CRC patients. More importantly, spearman correlation analysis revealed that GRIm-Score showed strong association with the well-established inflammatory indexes (SII, PNI, and ALRI). In brief, GRIm-Score correlated well with the established inflammatory scores, and exhibited superior predictive performance of prognosis in CRC patients compared with well-established inflammatory indexes.

PSM offers obvious advantages over traditional regression models to control for confounding factors in retrospective observational studies (30). Recently, PSM analysis is a very popular method to minimize the confounding effect due to measured covariates, as included subjects frequently differ from control individuals (31). In our study, given that the two groups (high GRIm-Score group vs. Low GRIm-Score group) dichotomized above were really not randomized, unbalanced variables might lead to selection bias. Hence, we utilized a 1:3 PSM method to complete the non-random assignment of CRC patients. The prognostic significance and predictive performance of GRIm-Score was more significant in the PSM cohort than that in the entire cohort. So, our conclusion based on PSM analysis was more rigorous and robust. Our results held great clinical significance in the risk stratification of postoperative patients with CRC. As CRC patients with high levels of preoperative GRIm-Score usually signify more unfavorable survival outcomes, so these patients may require more frequent follow-up and need more adjuvant chemotherapy or radiotherapy.

Several potential limitations in this study should not be ignored. Firstly, the clinical information was from a single-center retrospective cohort, and no external validation was further performed to verify our conclusions. Second, due to the nature of retrospective study, the value of GRIm-Score was preoperatively measured at a single time point rather than multiple time points. So, the dynamic prognostic significance of GRIm-Score was still unknown. Then, the predictive performance of GRIm-Score for the prediction of 1-year, 3-year, and 5-year OS and DFS was acceptable rather than good level. Going forward, prospective clinical studies regarding GRIm-Score and CRC patients will require larger external study populations to verify our conclusions.

## Conclusion

GRIm-Score, a novel inflammatory and nutritional risk scoring system, is a potent prognostic factor in CRC patients receiving surgical resection. The GRIm-Score can be used as an effective and simplified risk stratification tool for survival prediction of postoperative CRC patients.

## Data Availability Statement

The original contributions presented in the study are included in the article/**Supplementary Material**. Further inquiries can be directed to the corresponding authors.

## Ethics Statement

The studies involving human participants were reviewed and approved by the Clinical Research Ethics Committee of Wuhan Union Hospital (No. 2018-S377). The patients/participants provided their written informed consent to participate in this study.

## Author Contributions

PP and QL designed the research. ST and YC collected the clinical data. YD analyzed the data. ST wrote the manuscript. YC revised the manuscript. All authors contributed to the article and approved the submitted version.

## Funding

This study was supported by the National Natural Science Foundation of China (Nos.82060541, 82060111) and the Hubei Health Committee (No. WJ2019M102).

## Conflict of Interest

The authors declare that the research was conducted in the absence of any commercial or financial relationships that could be construed as a potential conflict of interest.

## Publisher’s Note

All claims expressed in this article are solely those of the authors and do not necessarily represent those of their affiliated organizations, or those of the publisher, the editors and the reviewers. Any product that may be evaluated in this article, or claim that may be made by its manufacturer, is not guaranteed or endorsed by the publisher.
