# Supplementary material for: Gustave Roussy Immune Score as a Novel Prognostic Scoring System for Colorectal Cancer Patients: A Propensity Score Matching Analysis
Source: Front Oncol. 2021 Nov 30;11:737283. doi: 10.3389/fonc.2021.737283 (PMC8669102; doi:10.3389/fonc.2021.737283)
Supplement: Supplementary file 1 [file DataSheet_1.pdf]

**Table S1**. CRC Patient characteristics between the four-category GRIm-Score of the entire cohort

| Features | | score 0 | score 1 | score 2 | score 3 | p value |
|---|---|---|---|---|---|---|
| Age | ≤60 | 540(56.19) | 245(58.61) | 95(53.98) | 13(54.17) | 0.7285 |
| | >60 | 421(43.81) | 173(41.39) | 81(46.02) | 11(45.83) | |
| Tumor size | ≤5 | 792(82.41) | 302(72.25) | 106(60.23) | 12(50.00) | <0.0001 |
| | >5 | 169(17.59) | 116(27.75) | 70(39.77) | 12(50.00) | |
| BMI | <18.5 | 53(5.53) | 29(6.94) | 22(12.57) | 1(4.17) | 0.0068 |
| | 18.5~24 | 570(59.50) | 274(65.55) | 102(58.29) | 17(70.83) | |
| | ≥24 | 335(34.97) | 115(27.51) | 51(29.14) | 6(25.00) | |
| TNM stage | I | 149(15.50) | 57(13.64) | 15(8.52) | 3(12.50) | 0.2265 |
| | II | 334(34.76) | 137(32.78) | 68(38.64) | 7(29.17) | |
| | III | 371(38.61) | 180(43.06) | 67(38.07) | 11(45.83) | |
| | IV | 107(11.13) | 44(10.53) | 26(14.77) | 3(12.50) | |
| T stage | T1 | 84(8.74) | 22(5.26) | 4(2.27) | 2(8.33) | 0.0002 |
| | T2 | 157(16.34) | 74(17.70) | 26(14.77) | 5(20.83) | |
| | T3 | 548(57.02) | 220(52.63) | 93(52.84) | 12(50.00) | |
| | T4 | 172(17.90) | 102(24.40) | 53(30.11) | 5(20.83) | |
| N stage | N0 | 569(59.21) | 236(56.46) | 88(50.00) | 13(54.17) | 0.2275 |
| | N1 | 240(24.97) | 110(26.32) | 55(31.25) | 6(25.00) | |
| | N2 | 152(15.82) | 72(17.22) | 33(18.75) | 5(20.83) | |
| Tumor site | left | 265(27.58) | 121(28.95) | 73(41.48) | 10(41.67) | <0.0001 |
| | right | 162(16.86) | 99(23.68) | 50(28.41) | 6(25.00) | |
| | rectum | 534(55.57) | 198(47.37) | 53(30.11) | 8(33.33) | |
| Diferentation | poor | 145(15.09) | 62(14.83) | 27(15.34) | 5(20.83) | 0.8651 |
| | moderate | 755(78.56) | 322(77.03) | 136(77.27) | 15(62.50) | |
| | well | 61(6.35) | 34(8.13) | 13(7.39) | 4(16.67) | |
| Gender | Male | 576(59.94) | 242(57.89) | 108(61.36) | 15(62.50) | 0.8349 |
| | female | 385(40.06) | 176(42.11) | 68(38.64) | 9(37.50) | |
| Smoke | No | 744(77.42) | 332(79.43) | 134(76.14) | 19(79.17) | 0.7945 |
| | Yes | 217(22.58) | 86(20.57) | 42(23.86) | 5(20.83) | |
| family cancer history | Yes | 109(11.34) | 34(8.13) | 9(5.11) | 0(0.00) | 0.0118 |
| | No | 852(88.66) | 384(91.87) | 167(94.89) | 24(100.00) | |
| Chemotherapy | No | 424(44.12) | 201(48.09) | 96(54.55) | 16(66.67) | 0.0113 |
| | Yes | 537(55.88) | 217(51.91) | 80(45.45) | 8(33.33) | |
| Radiotherapy | No | 902(93.86) | 400(95.69) | 168(95.45) | 23(95.83) | 0.5118 |
| | Yes | 59(6.14) | 18(4.31) | 8(4.55) | 1(4.17) | |
| LDH | ≤220 | 799(100.00) | 248(71.88) | 85(55.56) | 0(0.00) | <0.0001 |
| | >220 | 0(0.00) | 97(28.12) | 68(44.44) | 24(100.00) | |
| NLR | ≤3.68 | 961(100.00) | 210(50.24) | 15(8.52) | 0(0.00) | <0.0001 |
| | >3.68 | 0(0.00) | 208(49.76) | 161(91.48) | 24(100.00) | |
| PLR | ≤149.20 | 617(64.20) | 137(32.78) | 29(16.48) | 6(25.00) | <0.0001 |

| | | | | | | |
|---|---|---|---|---|---|---|
| | >149.20 | 344(35.80) | 281(67.22) | 147(83.52) | 18(75.00) | |
| MLR | ≤0.28 | 640(66.60) | 138(33.01) | 16(9.09) | 1(4.17) | <0.0001 |
| | >0.28 | 321(33.40) | 280(66.99) | 160(90.91) | 23(95.83) | |
| SII | ≤537.86 | 655(68.16) | 125(29.90) | 10(5.68) | 0(0.00) | <0.0001 |
| | >537.86 | 306(31.84) | 293(70.10) | 166(94.32) | 24(100.00) | |
| ALRI | ≤12.80 | 560(58.27) | 172(41.15) | 51(28.98) | 7(29.17) | <.0001 |
| | >12.80 | 401(41.73) | 246(58.85) | 125(71.02) | 17(70.83) | |
| PNI | ≤30.42 | 570(59.31) | 161(38.52) | 45(25.57) | 13(54.17) | <0.0001 |
| | >30.42 | 391(40.69) | 257(61.48) | 131(74.43) | 11(45.83) | |
| CEA | ≤3.60 | 521(54.27) | 194(46.63) | 79(45.93) | 7(29.17) | 0.0035 |
| | >3.60 | 439(45.73) | 222(53.37) | 93(54.07) | 17(70.83) | |
| CA724 | ≤2.12 | 411(50.24) | 185(50.00) | 82(53.95) | 4(20.00) | 0.043 |
| | >2.12 | 407(49.76) | 185(50.00) | 70(46.05) | 16(80.00) | |
| CA199 | ≤8.6 | 510(53.13) | 191(45.80) | 79(45.66) | 11(45.83) | 0.0438 |
| | >8.6 | 450(46.88) | 226(54.20) | 94(54.34) | 13(54.17) | |
| CA125 | ≤11.8 | 558(58.13) | 181(43.41) | 49(28.32) | 5(20.83) | <0.0001 |
| | >11.8 | 402(41.88) | 236(56.59) | 124(71.68) | 19(79.17) | |
| ALT | ≤40 | 885(92.09) | 386(92.34) | 162(92.05) | 23(95.83) | 0.9245 |
| | >40 | 76(7.91) | 32(7.66) | 14(7.95) | 1(4.17) | |
| AST | ≤40 | 922(95.94) | 392(93.78) | 165(93.75) | 22(91.67) | 0.2329 |
| | >40 | 39(4.06) | 26(6.22) | 11(6.25) | 2(8.33) | |
| ALB | >35 | 961(100.00) | 305(72.97) | 53(30.11) | 0(0.00) | <0.0001 |
| | ≤35 | 0(0.00) | 113(27.03) | 123(69.89) | 24(100.00) | |
| PAB | >0.2 | 396(62.96) | 101(38.55) | 21(17.80) | 1(7.14) | <0.0001 |
| | ≤0.2 | 233(37.04) | 161(61.45) | 97(82.20) | 13(92.86) | |
| TP | >64 | 578(60.15) | 205(49.04) | 46(26.14) | 2(8.33) | <0.0001 |
| | ≤64 | 383(39.85) | 213(50.96) | 130(73.86) | 22(91.67) | |
| DBIL | ≤6.8 | 801(91.33) | 311(84.74) | 128(80.00) | 16(69.57) | <0.0001 |
| | >6.8 | 76(8.67) | 56(15.26) | 32(20.00) | 7(30.43) | |
| TBIL | ≤19 | 866(90.11) | 362(86.60) | 155(88.07) | 19(79.17) | 0.1104 |
| | >19 | 95(9.89) | 56(13.40) | 21(11.93) | 5(20.83) | |
| URIC | ≤420/360 | 839(87.58) | 365(87.32) | 153(87.93) | 20(83.33) | 0.9346 |
| | >420/360 | 119(12.42) | 53(12.68) | 21(12.07) | 4(16.67) | |
| CREA | ≤133 | 948(98.96) | 412(98.56) | 169(97.13) | 24(100.00) | 0.2662 |
| | >133 | 10(1.04) | 6(1.44) | 5(2.87) | 0(0.00) | |
| BUN | ≤8.2 | 841(96.00) | 356(92.71) | 148(90.24) | 19(86.36) | 0.0026 |
| | >8.2 | 35(4.00) | 28(7.29) | 16(9.76) | 3(13.64) | |
| TBA | ≤10 | 686(90.86) | 283(92.18) | 121(90.98) | 14(87.50) | 0.8606 |
| | >10 | 69(9.14) | 24(7.82) | 12(9.02) | 2(12.50) | |
| GGT | ≤50 | 896(93.24) | 376(89.95) | 137(77.84) | 16(66.67) | <0.0001 |
| | >50 | 65(6.76) | 42(10.05) | 39(22.16) | 8(33.33) | |
| ALP | ≤150 | 951(98.96) | 408(97.61) | 168(95.45) | 22(91.67) | 0.0014 |
| | >150 | 10(1.04) | 10(2.39) | 8(4.55) | 2(8.33) | |

BMI: body mass index, NLR: neutrophil to lymphocyte ratio, PLR: platelet to lymphocyte ratio,

SII:systemic immune-inflammation index; ALRI: aspartate aminotransferase to lymphocyte ratio , PNI: prognostic nutritional index, CEA:carcinoembryonic antigen, ALT:alanine aminotransferase、AST:aspartate aminotransferase, ALB:albumin , PAB:prealbumin, TP:total protein, DBIL:direct bilirubin, TBIL: total bilirubin, GGT:glutamyl transpeptidase, ALP:alkaline phosphatase, LDH:lactate dehydrogenase, TBA:total bile acid, UA:uric acid (UA)、CREA: creatinine, BUN: blood urea nitrogen.

**Table S2**. Prognostic factors of overall survival in the entire cohort of CRC patients

| **Characteristics** | | **Univariable Analysis** | | **Multivariable Analysis** | |
|---|---|---|---|---|---|
| | | **HR with 95%CI** | ***P*-value** | **HR with 95%CI** | ***P*-value** |
| Age | ≤60 | **Reference** | | | |
| | >60 | 1.025(0.806~1.304) | 0.8382 | | |
| Tumor Size | ≤5 | **Reference** | | | |
| | >5 | 1.155(0.878~1.521) | 0.3038 | | |
| GRIm-SCORE | Score 0 | **Reference** | | **Reference** | |
| | Score 1 | 1.932(1.466~2.546) | <0.0001 | 1.548(1.067~2.247) | 0.0214 |
| | Score 2 | 2.561(1.841~3.561) | <0.0001 | 1.582(1.015~2.464) | 0.0426 |
| | Score 3 | 7.616(4.368~13.278) | <0.0001 | 8.620(4.111~18.072) | <0.0001 |
| sex | Male | **Reference** | | | |
| | Female | 0.927(0.725~1.184) | 0.5442 | | |
| smoke | Never | **Reference** | | **Reference** | |
| | Current/former | 0.612(0.439~0.854) | 0.0039 | 0.541(0.343~0.853) | 0.0082 |
| history | No | **Reference** | | | |
| | Yes | 1.425(0.883~2.298) | 0.1469 | | |
| Chemotherapy | No | **Reference** | | **Reference** | |
| | Yes | 0.589(0.462~0.752) | <.0001 | 0.586(0.423~0.812) | 0.0013 |
| Radiotherapy | No | **Reference** | | | |
| | Yes | 0.890(0.499~1.588) | 0.6931 | | |
| PLR | ≤149.20 | **Reference** | | | |
| | >149.20 | 1.557(1.220~1.987) | 0.0004 | | |
| SII_G | ≤537.86 | **Reference** | | | |
| | >537.86 | 1.745(1.363~2.232) | <.0001 | | |
| ALRI | ≤12.80 | **Reference** | | | |
| | >12.80 | 1.289(1.013~1.639) | 0.0386 | | |
| PNI | ≤30.42 | **Reference** | | | |
| | >30.42 | 1.058(0.834~1.344) | 0.6417 | | |
| CEA | ≤3.60 | **Reference** | | **Reference** | |
| | >3.60 | 2.426(1.878~3.134) | <.0001 | 1.639(1.169~2.299) | 0.0042 |
| CA724 | ≤2.12 | **Reference** | | | |
| | >2.12 | 1.467(1.132~1.901) | 0.0038 | | |
| CA199 | ≤8.6 | **Reference** | | | |
| | >8.6 | 1.822(1.422~2.335) | <.0001 | | |
| CA125 | ≤11.8 | **Reference** | | | |

| | | | | | |
|---|---|---|---|---|---|
| | >11.8 | 2.009(1.565~2.579) | <.0001 | | |
| ALT | ≤40 | **Reference** | | | |
| | >40 | 1.055(0.682~1.632) | 0.8092 | | |
| AST | ≤40 | **Reference** | | | |
| | >40 | 1.963(1.280~3.009) | 0.002 | | |
| PA | >0.2 | **Reference** | | **Reference** | |
| | ≤0.2 | 2.394(1.734~3.307) | <.0001 | 1.483(1.041~2.113) | 0.0292 |
| TP | >64 | **Reference** | | | |
| | ≤64 | 1.155(0.910~1.467) | 0.236 | | |
| DBIL | ≤6.8 | **Reference** | | | |
| | >6.8 | 1.442(1.031~2.018) | 0.0324 | | |
| TBIL | ≤19 | **Reference** | | | |
| | >19 | 1.307(0.924~1.849) | 0.1303 | | |
| URIC | ≤420/360 | **Reference** | | | |
| | >420/360 | 0.897(0.617~1.305) | 0.5705 | | |
| CREA | ≤133 | **Reference** | | | |
| | >133 | 2.044(0.965~4.329) | 0.0619 | | |
| BUN | ≤8.2 | **Reference** | | | |
| | >8.2 | 1.533(0.981~2.396) | 0.0608 | | |
| TBA | ≤10 | **Reference** | | | |
| | >10 | 1.175(0.741~1.865) | 0.4925 | | |
| GGT | ≤50 | **Reference** | | **Reference** | |
| | >50 | 2.613(1.939~3.522) | <.0001 | 2.382(1.557~3.645) | <.0001 |
| ALP | ≤150 | **Reference** | | **Reference** | |
| | >150 | 3.124(1.789~5.455) | <.0001 | 0.321(0.119~0.869) | 0.0253 |
| BMI | | | | | |
| | <18.5 | **Reference** | | | |
| | 18.5~24 | 0.712(0.468~1.083) | 0.1125 | | |
| | ≥24 | 0.524(0.332~0.826) | 0.0054 | | |
| TNM | | | | | |
| | I | **Reference** | | | |
| | II | 1.788(0.955~3.350) | 0.0696 | | |
| | III | 4.457(2.469~8.045) | <.0001 | | |
| | IV | 9.098(4.937~16.766) | <.0001 | | |
| T stage | | | | | |
| | T1 | **Reference** | | **Reference** | |
| | T2 | 1.144(0.481~2.721) | 0.7607 | 0.653(0.216~1.973) | 0.4498 |
| | T3 | 2.580(1.206~5.519) | 0.0146 | 2.396(0.882~6.510) | 0.0866 |
| | T4 | 7.305(3.404~15.674) | <.0001 | 4.027(1.456~11.137) | 0.0073 |
| N stage | | | | | |
| | N0 | **Reference** | | **Reference** | |
| | N1 | 2.325(1.739~3.110) | <.0001 | 2.204(1.257~3.862) | 0.0058 |
| | N2 | 4.405(3.287~5.902) | <.0001 | 3.381(1.935~5.908) | <.0001 |
| Tumor site | | | | | |

| | | | | | |
|---|---|---|---|---|---|
| | left | **Reference** | | | |
| | right | 1.061(0.770~1.464) | 0.716 | | |
| | rectum | 0.742(0.563~0.977) | 0.0339 | | |
| Differentiation | | | | | |
| | poor | **Reference** | | | |
| | moderate | 0.932(0.675~1.285) | 0.666 | | |
| | well | 0.603(0.325~1.117) | 0.108 | | |

BMI: body mass index, NLR: neutrophil to lymphocyte ratio, PLR: platelet to lymphocyte ratio, SII:systemic immune-inflammation index; ALRI: aspartate aminotransferase to lymphocyte ratio , PNI: prognostic nutritional index, CEA:carcinoembryonic antigen, ALT:alanine aminotransferase、AST:aspartate aminotransferase, ALB:albumin , PAB:prealbumin, TP:total protein, DBIL:direct bilirubin, TBIL: total bilirubin, GGT:glutamyl transpeptidase, ALP:alkaline phosphatase, LDH:lactate dehydrogenase, TBA:total bile acid, UA:uric acid (UA)、CREA: creatinine, BUN: blood urea nitrogen.

**Table S3**. Prognostic factors of disease-free survival in the entire cohort of CRC patients

| **Characteristics** | | **Univariable Analysis** | | **Multivariable Analysis** | |
|---|---|---|---|---|---|
| | | **HR with 95%CI** | ***P*-value** | **HR with 95%CI** | ***P*-value** |
| Age | ≤60 | **Reference** | | | |
| | >60 | 0.981(0.767~1.255) | 0.8804 | | |
| Size | ≤5 | **Reference** | | | |
| | >5 | 0.987(0.738~1.319) | 0.9273 | | |
| GRIm-Score | Score 0 | **Reference** | | **Reference** | |
| | Score 1 | 1.794(1.351~2.384) | <0.0001 | 1.886(1.317~2.701) | 0.0005 |
| | Score 2 | 2.466(1.770~3.436) | <0.0001 | 2.016(1.291~3.148) | 0.002 |
| | Score 3 | 7.174(4.113~12.513) | <0.0001 | 10.99(5.185~23.309) | <0.0001 |
| sex | Male | **Reference** | | | |
| | Female | 1.036(0.809~1.327) | 0.7794 | | |
| smoke | Never | **Reference** | | **Reference** | |
| | Yes | 0.579(0.409~0.820) | 0.0021 | 0.617(0.384~0.991) | 0.0459 |
| Family history of cancer | No | **Reference** | | | |
| | Yes | 1.102(0.705~1.723) | 0.6687 | | |
| Chemotherapy | No | **Reference** | | | |
| | Yes | 0.960(0.753~1.225) | 0.7453 | | |
| Radiotherapy | No | **Reference** | | | |
| | Yes | 1.652(1.046~2.607) | 0.0312 | | |
| PLR | ≤149.20 | **Reference** | | | |
| | >149.20 | 1.528(1.192~1.960) | 0.0008 | | |
| SII | ≤537.86 | **Reference** | | | |
| | >537.86 | 1.745(1.355~2.246) | <0.0001 | | |
| ALRI | ≤12.80 | **Reference** | | | |

| | | | | | |
|---|---|---|---|---|---|
| | >12.80 | 1.237(0.968~1.580) | 0.0886 | | |
| PNI | ≤30.42 | **Reference** | | | |
| | >30.42 | 1.142(0.895~1.457) | 0.2859 | | |
| CEA | ≤3.60 | **Reference** | | | |
| | >3.60 | 1.773(1.379~2.279) | <0.0001 | | |
| CA724 | ≤2.12 | **Reference** | | | |
| | >2.12 | 1.448(1.113~1.883) | 0.0058 | | |
| CA199 | ≤8.6 | **Reference** | | | |
| | >8.6 | 1.749(1.359~2.252) | <0.0001 | | |
| CA125 | ≤11.8 | **Reference** | | | |
| | >11.8 | 1.669(1.299~2.144) | <0.0001 | | |
| ALT | ≤40 | **Reference** | | | |
| | >40 | 1.187(0.780~1.807) | 0.423 | | |
| AST | ≤40 | **Reference** | | **Reference** | |
| | >40 | 2.023(1.318~3.104) | 0.0013 | 1.875(1.050~3.350) | 0.0337 |
| PA | >0.2 | **Reference** | | | |
| | ≤0.2 | 1.877(1.371~2.569) | <0.0001 | | |
| TP | >64 | **Reference** | | | |
| | ≤64 | 1.079(0.846~1.377) | 0.5395 | | |
| DBIL | ≤6.8 | **Reference** | | | |
| | >6.8 | 1.321(0.934~1.869) | 0.1159 | | |
| TBIL | ≤19 | **Reference** | | | |
| | >19 | 1.153(0.797~1.670) | 0.4498 | | |
| URIC | ≤420/360 | **Reference** | | | |
| | >420/360 | 0.786(0.527~1.171) | 0.2362 | | |
| CREA | ≤133 | **Reference** | | | |
| | >133 | 1.788(0.796~4.017) | 0.1596 | | |
| BUN | ≤8.2 | **Reference** | | | |
| | >8.2 | 1.044(0.620~1.760) | 0.8702 | | |
| TBA | ≤10 | **Reference** | | | |
| | >10 | 1.207(0.760~1.916) | 0.4256 | | |
| GGT | ≤50 | **Reference** | | | |
| | >50 | 2.392(1.764~3.243) | <0.0001 | | |
| ALP | ≤150 | **Reference** | | | |
| | >150 | 3.017(1.725~5.275) | 0.0001 | | |
| BMI | | | | | |
| | <18.5 | **Reference** | | | |
| | 18.5~24 | 0.722(0.462~1.129) | 0.1531 | | |
| | ≥24 | 0.694(0.433~1.111) | 0.1278 | | |
| TNM | | | | | |
| | I | **Reference** | | **Reference** | |
| | II | 2.556(1.151~5.674) | 0.0211 | 0.899(0.290~2.785) | 0.8535 |
| | III | 5.727(2.664~12.313) | <0.0001 | 1.464(0.469~4.576) | 0.5118 |
| | IV | 25.055(11.655~53.864 | <0.0001 | 6.116(1.992~18.778) | 0.0016 |

| | | | | | |
|---|---|---|---|---|---|
| T stage | | | | | |
| | T1 | **Reference** | | **Reference** | |
| | T2 | 1.982(0.674~5.826) | 0.2137 | 0.852(0.252~2.879) | 0.7971 |
| | T3 | 4.665(1.725~12.613) | 0.0024 | 2.923(1.024~8.339) | 0.045 |
| | T4 | 10.763(3.964~29.226) | <0.0001 | 3.415(1.167~9.993) | 0.025 |
| N stage | | | | | |
| | N0 | **Reference** | | **Reference** | |
| | N1 | 2.076(1.540~2.800) | <0.0001 | 1.404(0.845~2.332) | 0.1897 |
| | N2 | 4.211(3.141~5.646) | <0.0001 | 2.077(1.283~3.362) | 0.0029 |
| Tumor site | | | | | |
| | left | **Reference** | | | |
| | right | 1.004(0.721~1.397) | 0.9823 | | |
| | rectum | 0.777(0.588~1.027) | 0.0763 | | |
| Differentiation | | | | | |
| | poor | **Reference** | | | |
| | moderate | 1.191(0.832~1.705) | 0.339 | | |
| | well | 1.180(0.681~2.045) | 0.5546 | | |

BMI: body mass index, NLR: neutrophil to lymphocyte ratio, PLR: platelet to lymphocyte ratio, SII:systemic immune-inflammation index; ALRI: aspartate aminotransferase to lymphocyte ratio , PNI: prognostic nutritional index, CEA:carcinoembryonic antigen, ALT:alanine aminotransferase、AST:aspartate aminotransferase, ALB:albumin , PAB:prealbumin, TP:total protein, DBIL:direct bilirubin, TBIL: total bilirubin, GGT:glutamyl transpeptidase, ALP:alkaline phosphatase, LDH:lactate dehydrogenase, TBA:total bile acid, UA:uric acid (UA)、CREA: creatinine, BUN: blood urea nitrogen.

**Table S4.** Comparision of CRC patients between the low and high GRIm-Score groups in the whole cohort

| Clinical feature | | Low group N(%) | High group N(%) | $X^2$ | P value |
|---|---|---|---|---|---|
| Age (years) | ≤60 | 785(56.93) | 108(54.00) | 0.608 | 0.4356 |
| | >60 | 594(43.07) | 92(46.00) | | |
| Gender | Male | 818(59.32) | 123(61.50) | 0.345 | 0.5569 |
| | Female | 561(40.68) | 77(38.50) | | |
| Smoke | No | 1076(78.03) | 153(76.50) | 0.236 | 0.627 |
| | Yes | 303(21.97) | 47(23.50) | | |
| Family history of cancer | Yes | 143(10.37) | 9(4.50) | 6.913 | 0.0086 |
| | No | 1236(89.63) | 191(95.50) | | |
| BMI ($Kg/m^2$) | <18.5 | 82(5.96) | 23(11.56) | 5.012 | 0.0252 |

| | | | | | |
|---|---|---|---|---|---|
| | 18.5~24 | 844(61.34) | 119(59.80) | | |
| | ≥24 | 450(32.70) | 57(28.64) | | |
| Tumor stage | T1 | 106(7.69) | 6(3.00) | 10.474 | 0.0012 |
| | T2 | 231(16.75) | 31(15.50) | | |
| | T3 | 768(55.69) | 105(52.50) | | |
| | T4 | 274(19.87) | 58(29.00) | | |
| N stage | N0 | 805(58.38) | 101(50.50) | 3.441 | 0.0636 |
| | N1 | 350(25.38) | 61(30.50) | | |
| | N2 | 224(16.24) | 38(19.00) | | |
| TNM stage | I | 206(14.94) | 18(9.00) | 3.346 | 0.0674 |
| | II | 471(34.16) | 75(37.50) | | |
| | III | 551(39.96) | 78(39.00) | | |
| | IV | 151(10.95) | 29(14.50) | | |
| Tumor site | Right | 386(27.99) | 83(41.50) | 30.03 | <0.0001 |
| | Left | 261(18.93) | 56(28.00) | | |
| | Rectum | 732(53.08) | 61(30.50) | | |
| Differentiation | Low | 207(15.01) | 32(16.00) | 0.031 | 0.8596 |
| | Moderate | 1077(78.10) | 151(75.50) | | |
| | High | 95(6.89) | 17(8.50) | | |
| Tumor size (cm) | ≤5 | 1094(79.33) | 118(59.00) | 40.451 | <0.0001 |
| | >5 | 285(20.67) | 82(41.00) | | |
| Chemotherapy | No | 625(45.32) | 112(56.00) | 7.995 | 0.0047 |
| | Yes | 754(54.68) | 88(44.00) | | |
| Radiotherapy | No | 1302(94.42) | 191(95.50) | 0.398 | 0.5281 |
| | Yes | 77(5.58) | 9(4.50) | | |
| NLR | ≤3.68 | 1171(84.92) | 15(7.50) | 559.62 | <0.0001 |
| | >3.68 | 208(15.08) | 185(92.50) | | |
| PLR | ≤149.20 | 754(54.68) | 35(17.50) | 96.506 | <0.0001 |
| | >149.20 | 625(45.32) | 165(82.50) | | |
| SII | ≤537.86 | 780(56.56) | 10(5.00) | 185.64 | <0.0001 |
| | >537.86 | 599(43.44) | 190(95.00) | | |
| ALRI | ≤12.80 | 732(53.08) | 58(29.00) | 40.493 | <0.0001 |
| | >12.80 | 647(46.92) | 142(71.00) | | |
| PNI | ≤30.42 | 731(53.01) | 58(29.00) | 40.25 | <0.0001 |
| | >30.42 | 648(46.99) | 142(71.00) | | |
| CEA (ug/L) | ≤3.60 | 715(51.96) | 86(43.88) | 4.484 | 0.0342 |
| | >3.60 | 661(48.04) | 110(56.12) | | |
| CA724 (U/mol) | ≤2.12 | 596(50.17) | 86(50.00) | 0.002 | 0.9671 |
| | >2.12 | 592(49.83) | 86(50.00) | | |
| CA199 (U/ml) | ≤8.6 | 701(50.91) | 90(45.69) | 1.879 | 0.1704 |
| | >8.6 | 676(49.09) | 107(54.31) | | |
| CA125 (U/ml) | ≤11.8 | 739(53.67) | 54(27.41) | 47.497 | <0.0001 |
| | >11.8 | 638(46.33) | 143(72.59) | | |
| ALT(U/L) | ≤40 | 1271(92.17) | 185(92.50) | 0.027 | 0.8701 |

| | | | | | |
|---|---|---|---|---|---|
| | >40 | 108(7.83) | 15(7.50) | | |
| AST(U/L) | ≤40 | 1314(95.29) | 187(93.50) | 1.186 | 0.2761 |
| | >40 | 65(4.71) | 13(6.50) | | |
| ALB(g/L) | >35 | 1266(91.81) | 53(26.50) | 541.23 | <0.0001 |
| | ≤35 | 113(8.19) | 147(73.50) | | |
| PA(g/L) | >0.2 | 497(55.78) | 22(16.67) | 70.3 | <0.0001 |
| | ≤0.2 | 394(44.22) | 110(83.33) | | |
| TP (g/L) | >64 | 783(56.78) | 48(24.00) | 75.236 | <0.0001 |
| | ≤64 | 596(43.22) | 152(76.00) | | |
| DBIL(umol/L) | ≤6.8 | 1112(89.39) | 144(78.69) | 17.307 | <0.0001 |
| | >6.8 | 132(10.61) | 39(21.31) | | |
| TBIL (umol/L) | ≤19 | 1228(89.05) | 174(87.00) | 0.737 | 0.3906 |
| | >19 | 151(10.95) | 26(13.00) | | |
| UA (umol/L) | ≤420/360 | 1204(87.50) | 173(87.37) | 0.003 | 0.96 |
| | >420/360 | 172(12.50) | 25(12.63) | | |
| CREA (umol/L) | ≤133 | 1360(98.84) | 193(97.47) | 2.439 | 0.1183 |
| | >133 | 16(1.16) | 5(2.53) | | |
| BUN(mmol/L) | ≤8.2 | 1197(95.00) | 167(89.78) | 8.235 | 0.0041 |
| | >8.2 | 63(5.00) | 19(10.22) | | |
| TBA (umol/L) | ≤10 | 969(91.24) | 135(90.60) | 0.066 | 0.797 |
| | >10 | 93(8.76) | 14(9.40) | | |
| GGT(U/L) | ≤50 | 1272(92.24) | 153(76.50) | 49.138 | <0.0001 |
| | >50 | 107(7.76) | 47(23.50) | | |
| ALP (U/L) | ≤150 | 1359(98.55) | 190(95.00) | 11.801 | 0.0006 |
| | >150 | 20(1.45) | 10(5.00) | | |
| LDH (U/L) | ≤220 | 1047(91.52) | 85(48.02) | 236.38 | <0.0001 |
| | >220 | 97(8.48) | 92(51.98) | | |

BMI: body mass index, NLR: neutrophil to lymphocyte ratio, PLR: platelet to lymphocyte ratio, SII:systemic immune-inflammation index; ALRI: aspartate aminotransferase to lymphocyte ratio , PNI: prognostic nutritional index, CEA:carcinoembryonic antigen, ALT:alanine aminotransferase、AST:aspartate aminotransferase, ALB:albumin , PAB:prealbumin, TP:total protein, DBIL:direct bilirubin, TBIL: total bilirubin, GGT:glutamyl transpeptidase, ALP:alkaline phosphatase, LDH:lactate dehydrogenase, TBA:total bile acid, UA:uric acid (UA)、CREA: creatinine, BUN: blood urea nitrogen.

**Table S5.** Comparision of CRC patients between the low and high GRIm-Score groups in propensity-matched cohort

| Clinical feature | | Low group N(%) | High group N(%) | $X^2$ | P value |
|---|---|---|---|---|---|
| Age (years) | ≤60 | 310(55.66) | 108(54.55) | 0.073 | 0.7874 |
| | >60 | 247(44.34) | 90(45.45) | | |

| | | | | | |
|---|---|---|---|---|---|
| Gender | Male | 318(57.09) | 122(61.62) | 1.228 | 0.2677 |
| | Female | 239(42.91) | 76(38.38) | | |
| Smoke | No | 424(76.12) | 151(76.26) | 0.002 | 0.9682 |
| | Yes | 133(23.88) | 47(23.74) | | |
| Family history of cancer | Yes | 25(4.49) | 9(4.55) | 0.001 | 0.9735 |
| | No | 532(95.51) | 189(95.45) | | |
| BMI (Kg/m$^2$) | <18.5 | 44(7.90) | 23(11.62) | 0.032 | 0.8577 |
| | 18.5~24 | 378(67.86) | 118(59.60) | | |
| | ≥24 | 135(24.24) | 57(28.79) | | |
| T stage | T1 | 20(3.59) | 6(3.03) | 0.274 | 0.6008 |
| | T2 | 82(14.72) | 30(15.15) | | |
| | T3 | 306(54.94) | 104(52.53) | | |
| | T4 | 149(26.75) | 58(29.29) | | |
| N stage | N0 | 295(52.96) | 101(51.01) | 0.035 | 0.8512 |
| | N1 | 143(25.67) | 61(30.81) | | |
| | N2 | 119(21.36) | 36(18.18) | | |
| TNM stage | I | 53(9.52) | 17(8.59) | 0.007 | 0.9348 |
| | II | 196(35.19) | 75(37.88) | | |
| | III | 237(42.55) | 78(39.39) | | |
| | IV | 71(12.75) | 28(14.14) | | |
| Tumor site | Right | 234(42.01) | 82(41.41) | 0.387 | 0.5339 |
| | Left | 126(22.62) | 56(28.28) | | |
| | Rectum | 197(35.37) | 60(30.30) | | |
| Differentiation | Low | 74(13.29) | 32(16.16) | 0.554 | 0.4568 |
| | Moderate | 438(78.64) | 150(75.76) | | |
| | High | 45(8.08) | 16(8.08) | | |
| Tumor size (cm) | ≤5 | 346(62.12) | 118(59.60) | 0.392 | 0.5313 |
| | >5 | 211(37.88) | 80(40.40) | | |
| Chemotherapy | No | 284(50.99) | 110(55.56) | 1.22 | 0.2694 |
| | Yes | 273(49.01) | 88(44.44) | | |
| Radiotherapy | No | 529(94.97) | 190(95.96) | 0.313 | 0.5761 |
| | Yes | 28(5.03) | 8(4.04) | | |
| NLR | ≤3.68 | 459(82.41) | 14(7.07) | 353.81 | <0.0001 |
| | >3.68 | 98(17.59) | 184(92.93) | | |
| PLR | ≤149.20 | 274(49.19) | 35(17.68) | 59.93 | <0.0001 |
| | >149.20 | 283(50.81) | 163(82.32) | | |
| SII | ≤537.86 | 274(49.19) | 10(5.05) | 121.13 | <0.0001 |
| | >537.86 | 283(50.81) | 188(94.95) | | |
| ALRI | ≤12.80 | 301(54.04) | 58(29.29) | 35.821 | <0.0001 |
| | >12.80 | 256(45.96) | 140(70.71) | | |
| PNI | ≤30.42 | 297(53.32) | 57(28.79) | 35.258 | <0.0001 |
| | >30.42 | 260(46.68) | 141(71.21) | | |
| CEA (ug/L) | ≤3.60 | 256(46.04) | 86(44.33) | 0.17 | 0.6802 |

| | | | | | |
|---|---|---|---|---|---|
| | >3.60 | 300(53.96) | 108(55.67) | | |
| CA724 (U/mol) | ≤2.12 | 241(49.79) | 85(50.00) | 0.002 | 0.9631 |
| | >2.12 | 243(50.21) | 85(50.00) | | |
| CA199 (U/ml) | ≤8.6 | 245(44.06) | 90(46.15) | 0.255 | 0.6138 |
| | >8.6 | 311(55.94) | 105(53.85) | | |
| CA125 (U/ml) | ≤11.8 | 264(47.48) | 54(27.69) | 23.128 | <0.0001 |
| | >11.8 | 292(52.52) | 141(72.31) | | |
| ALT(U/L) | ≤40 | 522(93.72) | 184(92.93) | 0.149 | 0.6996 |
| | >40 | 35(6.28) | 14(7.07) | | |
| AST(U/L) | ≤40 | 531(95.33) | 185(93.43) | 1.073 | 0.3004 |
| | >40 | 26(4.67) | 13(6.57) | | |
| ALB(g/L) | >35 | 500(89.77) | 53(26.77) | 295.45 | <0.0001 |
| | ≤35 | 57(10.23) | 145(73.23) | | |
| PA(g/L) | >0.2 | 165(46.48) | 22(16.79) | 35.547 | <0.0001 |
| | ≤0.2 | 190(53.52) | 109(83.21) | | |
| TP (g/L) | >64 | 299(53.68) | 48(24.24) | 50.9 | <.0001 |
| | ≤64 | 522(93.72) | 184(92.93) | 0.149 | 0.6996 |
| DBIL(umol/L) | ≤6.8 | 441(88.02) | 143(78.57) | 9.61 | 0.0019 |
| | >6.8 | 60(11.98) | 39(21.43) | | |
| TBIL (umol/L) | ≤19 | 495(88.87) | 172(86.87) | 0.567 | 0.4515 |
| | >19 | 62(11.13) | 26(13.13) | | |
| UA (umol/L) | ≤420 (M)/360 | 487(87.75) | 171(87.24) | 0.034 | 0.8543 |
| | >420/360(F) | 68(12.25) | 25(12.76) | | |
| CREA (umol/L) | ≤133 | 551(99.28) | 191(97.45) | 4.093 | 0.0431 |
| | >133 | 4(0.72) | 5(2.55) | | |
| BUN(mmol/L) | ≤8.2 | 484(94.90) | 166(90.22) | 4.99 | 0.0255 |
| | >8.2 | 26(5.10) | 18(9.78) | | |
| TBA (umol/L) | ≤10 | 379(90.02) | 134(90.54) | 0.033 | 0.8561 |
| | >10 | 42(9.98) | 14(9.46) | | |
| GGT(U/L) | ≤50 | 507(91.02) | 152(76.77) | 26.712 | <0.0001 |
| | >50 | 50(8.98) | 46(23.23) | | |
| ALP (U/L) | ≤150 | 545(97.85) | 188(94.95) | 4.325 | 0.0376 |
| | >150 | 12(2.15) | 10(5.05) | | |
| LDH (U/L) | ≤220 | 423(92.76) | 85(48.30) | 158.97 | <0.0001 |
| | >220 | 33(7.24) | 91(51.70) | | |

BMI: body mass index, NLR: neutrophil to lymphocyte ratio, PLR: platelet to lymphocyte ratio, SII:systemic immune-inflammation index; ALRI: aspartate aminotransferase to lymphocyte ratio , PNI: prognostic nutritional index, CEA:carcinoembryonic antigen, ALT:alanine aminotransferase、AST:aspartate aminotransferase, ALB:albumin , PAB:prealbumin, TP:total protein, DBIL:direct bilirubin, TBIL: total bilirubin, GGT:glutamyl transpeptidase, ALP:alkaline phosphatase, LDH:lactate dehydrogenase, TBA:total bile acid, UA:uric acid (UA)、CREA: creatinine, BUN: blood urea nitrogen.

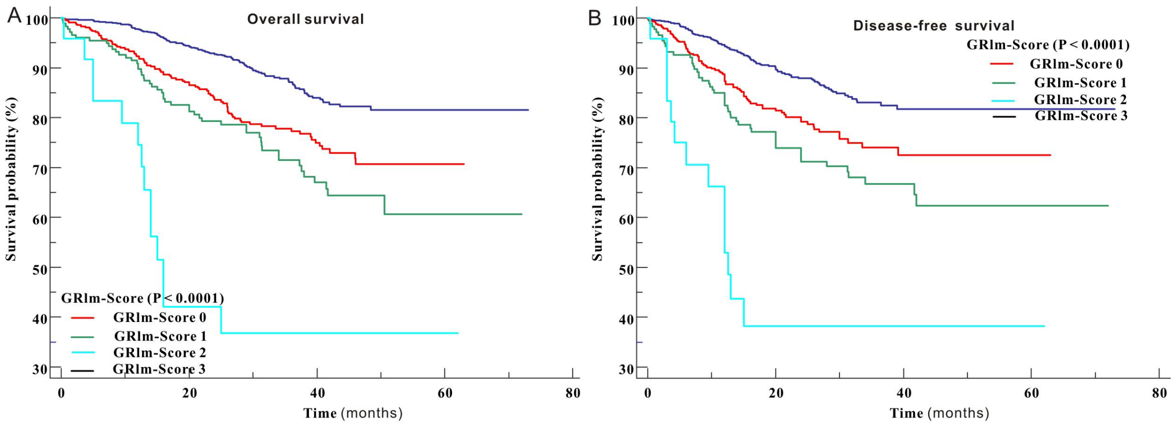


**Figure S1**: Prognostic significance of GRIm-score by four categories in patients with CRC. **A**. Overall survival, **B**. Disease-free survival.

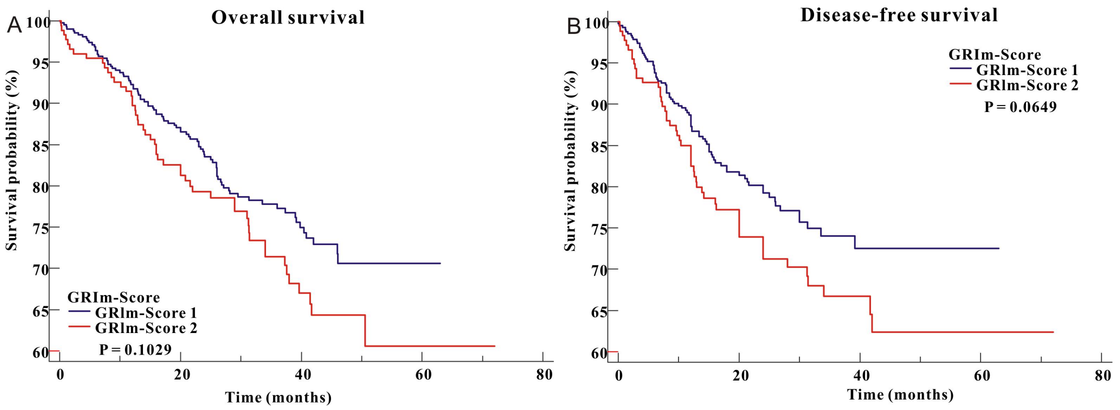


**Figure S2**: Survival difference of CRC patients with score 1 and patients with score 2. **A**. Overall survival, **B**. Disease-free survival.

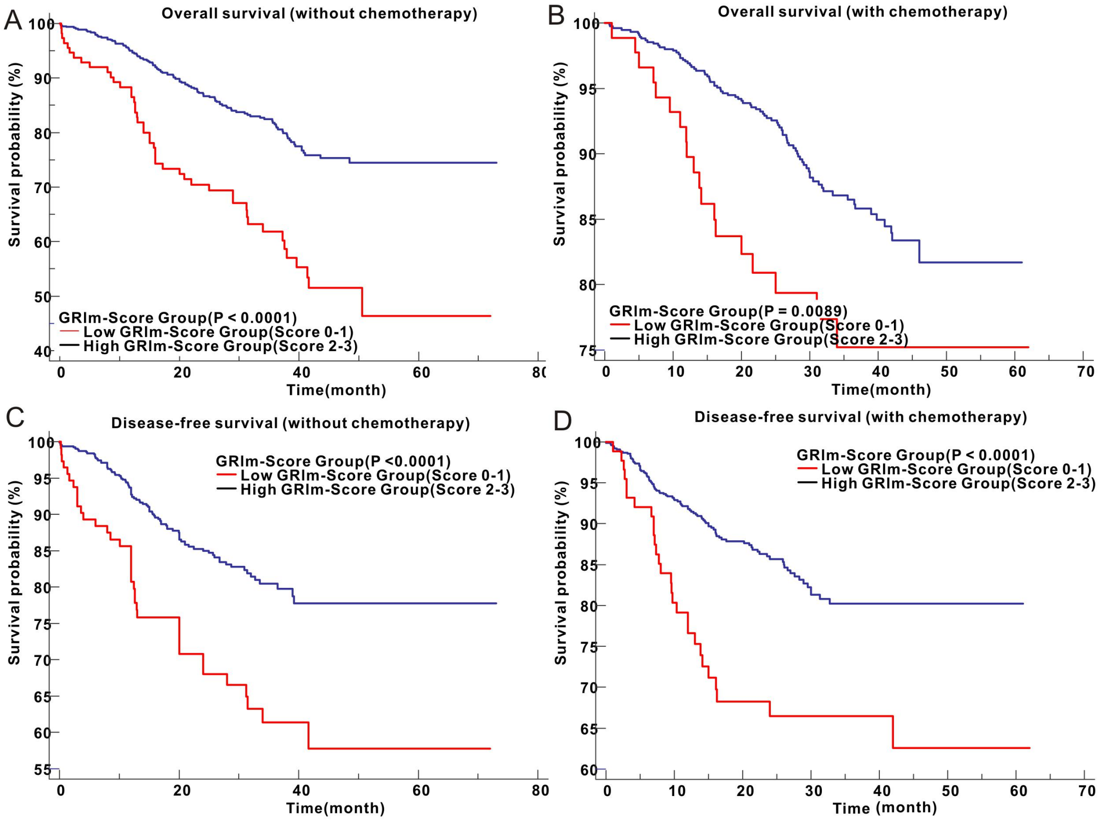


**Figure S3**. Survival analysis stratified based on chemotherapy. CRC patients with low GRIm-Score exhibited relatively longer overall survival time than those with high GRIm-Score both in individuals without chemotherapy (**A**) and with chemotherapy (**B**). CRC patients with low GRIm-Score exhibited relatively longer disease-free survival time than those with high GRIm-Score both in individuals without chemotherapy (**C**) and with chemotherapy (**D**).
